# Healthy eating and physical activity among breastfeeding women: the role of misinformation

**DOI:** 10.1186/s12884-020-03153-x

**Published:** 2020-08-17

**Authors:** Kailey Snyder, Aja Kneip Pelster, Danae Dinkel

**Affiliations:** 1 School of Pharmacy and Health Sciences, Creighton University, 2500 California Plaza, Omaha, NE 68178 USA; 2 Health Behavior, School of Health & Kinesiology, University of Nebraska at Omaha, 6001, Dodge Street, Omaha, NE 68182 USA; 3 Physical Activity in Health Promotion, School of Health & Kinesiology, University of Nebraska at Omaha, 6001Dodge Street, Omaha, NE 68182 USA

**Keywords:** Physical activity, Nutrition, Breastfeeding, Postpartum, Qualitative

## Abstract

**Background:**

Eating healthy, being physically active and breastfeeding can greatly support a new mother’s physiological and psychological health. However, within the United States, only 8.5% of women are meeting fruit and vegetable recommendations and only 35% of women are maintaining their pre-pregnancy physical activity after childbirth. Preliminary investigations suggest barriers such as lack of time and knowledge hinder a breastfeeding woman’s ability to eat well and be physically active. The purpose of this study was to explore women’s perceptions of healthy eating and physical activity while breastfeeding.

**Methods:**

This qualitative study utilized a 33-question semi-structured interview guide developed using the constructs of Self-Determination Theory. Data were analyzed via the process of immersion/crystallization.

**Results:**

A total of 24 breastfeeding women completed a roughly 40-min telephone interview. The mean age of the mother was 32 ± .88 and the mean age of the child being breastfed was 8.5 ± 1.4 months old. Findings demonstrate mothers see value in engaging in physical activity primarily for reasons related to self-care. In addition, breastfeeding women have a lack of information and support regarding healthy eating and physical activity while breastfeeding and many are receiving misinformation through resources such as *Facebook* support groups. Finally, mothers want more resources available that focus specifically on healthy behaviors while breastfeeding.

**Conclusion:**

Women need greater access to education and resources regarding healthy eating and physical activity while breastfeeding. Ideally, information and resources would come from an educated health professional such as their healthcare provider rather than an internet source.

## Background

A healthy diet and regular physical activity during the first year after childbirth (postpartum period) are essential to a woman’s health [1–5].^.^ Specific to dietary habits, a healthy diet can assist in postpartum weight loss as well prevent micronutrient deficiencies linked to postpartum depression [2, 3]. Unfortunately 6 months after childbirth less than 30% of women are eating the recommended amounts of fruits and vegetables [4]. In addition, physical activity in the postpartum period can improve a mother’s mood, stress and energy levels however the majority are not meeting physical activity recommendations [1, 5]. Not only does the general population struggle to achieve these healthy behaviors but breastfeeding women appear to suffer from additional barriers [5].^.^ This is concerning as breastfeeding provides substantial health benefits to mothers and their children and thus mothers physical health should be better supported while breastfeeding [6–8]. More research is needed to explore the health behaviors of breastfeeding women to determine how to improve support.

Two studies have been conducted to explore the health behaviors of breastfeeding women [9, 10]. A mixed methods study found women (*n* = 6) to be motivated for exercise by stress relief and energy improvement however women were concerned that exercise negatively influenced milk supply [9]. This study was limited by its small sample size and questions were only focused on exercise. Another study determined low-income mothers were motivated to engage in healthy behaviors like physical activity and healthy eating if it benefited their child, but they were not seeking out information regarding how to do so [10]. While this study is a crucial first step in this important work, it is limited by focusing only on low-income women and women were not required to be breastfeeding at the time of study participation, thus the results may have been based on recall experiences. More qualitative research is needed to better understand the barriers breastfeeding women face and what they feel they need to improve their dietary choices and physical activity behaviors [11]. Thus, the aim of this study was to explore women’s perceptions of healthy eating and physical activity while breastfeeding. Preliminary findings related to the physical activity findings of this study were shared previously and details can be found elsewhere [12].

## Methods

### Design

A phenomenological cross-sectional qualitative design guided by the self-determination theory (SDT) was taken to understand women’s experience engaging in healthy eating and physical activity while breastfeeding. Specifically, the study focused on the SDT constructs of motivation, autonomy, competence and relatedness [13]. A university institutional review board approved the study.

### Setting

Data were collected in the spring/summer of 2018 from women residing in Nebraska, United States in rural and urban areas. To differentiate rural and urban residents, census tract-based rural urban commuting area (RUCA) codes were utilized. Urban residents were defined as RUCA codes 1–6 and rural residencies codes 7–10 [14]. Women were recruited for survey participation in person via local breastfeeding support events in a large metro city in Nebraska as well as online via *Facebook* and the state’s breastfeeding coalition website. The state’s breastfeeding website has a reach across the state of Nebraska to allow for greater geographic diversity in subject recruitment as there are currently 400+ members.

### Sample

Women were included in the study if they were currently providing breastmilk to a child through pumping or breastfeeding and were at least 19, the legal age of adulthood in the state that the research took place. Women were recruited for the interview based on interest expressed in a survey completed on physical activity while breastfeeding [8]. They were told they would be participating in an interview about their perceptions engaging in healthy eating and physical activity while breastfeeding. A total of 35 women were contacted before 24 interviews were completed. Three scheduled interviews were cancelled and 8 women did not respond to initial contacting.

### Data collection

Women were contacted via their preferred method of communication as identified in the initial survey they completed. The moderator did not have a relationship with any of the participants prior to the interview being completed however did inform the women that she herself was a currently breastfeeding mother. All interviews took place over the phone and took approximately 40 min to complete. Women were asked to engage in the call in a private room free from other adults although their children were allowed to be present. The moderator, a PhD-candidate, extensively trained in qualitative methods had experience conducting over 150 interviews conducted all the interviews. Prior to the interviews being complete, the moderator provided a narrative of the interview process to the participant and asked them to respond yes/no if they understood what would be taking place during the phone call and if they would still like to participate. If they responded yes, the interview began.

The 33-question semi-structured interview guide was developed by the first author utilizing constructs of SDT [13]. Based on results from the survey, additional questions pertaining to the mother’s relationship with her healthcare provider and the provider’s promotion of breastfeeding and health behaviors were included. A draft of the interview guide was reviewed by two additional qualitative experts prior to being deemed final. A total of 51 women (29 urban; 22 rural) were contacted until the 24 interviews were completed. At this point data was analyzed by the first author to ensure saturation had been reached [14]. All interviews occurred over the phone and were audio recorded. The average interview lasted approximately 40 min. Example interview questions can be seen in Table 1.

**Table 1:** Semi-Structured Interview Question Examples

*SEM* Construct	Interview Question
Motivation	1. What motivates you to try to be healthy?
Autonomy	2. Has anyone ever told you to be physically active? Who? a. Did they ever give advice specific to being physically active while breastfeeding?
Relatedness	3. Would you say your health behaviors are similar to that of your friends? How so? a. Your family? How so?
Competence	4. What do you feel unsure of in regard to trying to engage in healthy behaviors while breastfeeding?

### Data analysis

The moderator being a currently breastfeeding female was selected in an attempt to allow the researcher to better connect to the respondents and elicit greater depth of personal experiences [15]. However, throughout the interview process the author maintained a reflexivity journal in which she wrote out her assumptions and biases to try and remain cognizant of them throughout the interviews [16, 17].

Upon completion of the interviews, the audio recordings were immediately transcribed verbatim and uploaded into NVivo 12, a qualitative analysis software [18]. In order to utilize appropriate terminology most effectively for the population a bottom up approach was taken to attempt to enhance the relevance of the results [19].

The bottom up analysis followed the steps developed by Braun and Clark [20]. The process began with the first author reading all the transcripts three times and annotating any significant or interesting statements found. The author then reviewed the transcripts a fourth time and marked any emerging themes that had evolved from initial field notes. The emergent themes for each transcript were then compiled into a single list to look for connections between one another. As clustering of themes emerged three main themes were identified with subthemes for each [21].

In addition, the author returned to her reflexivity journal to determine if any biases were influencing the analysis [22]. The first author then met with the last author to discuss these biases. The first author provided the other researcher with a document highlighting her prejudices and assumptions for this subject matter and allowed them to be discussed and challenged. The last author then reviewed the data to see if these biases were prevalent in the analysis. If a theme or sub-theme was suspected of bias, member checking was executed. This involved the first author returning to the quoted participant to determine if the results found accurately reflect their experiences [23]. After member checking was attempted, the first author once again met with the last author to discuss the themes and subthemes. After consensus was reached and it was confirmed the first author’s biases were limited as much as possible, peer debriefing was conducted with the second author who was a trained qualitative researcher but unfamiliar with breastfeeding and physical activity as an area of research [24].

## Results

### Participant characteristics

Subjects age ranged between 21 and 40 with a mean age of 32 (*SD = .88)*. The majority of women had at least a Bachelor’s degree or higher (75.0%) and were employed either part-time or full-time (66.6%). Half of women resided in a rural residence (< 20,000 population) and half within an urban residence. Children being provided breast milk ranged in age from 3 weeks to 20 months with a mean age of 8.5 months (*SD* = 1.14). For further participant details see Table 2.

**Table 2:** Sociodemographics of Participants

*Characteristic*	n *(%)*
*Age, years*	
19–24	6 (25.0)
25–30	7 (29.2)
31–35	12 (50.0)
36–40	4 (16.7)
*Education*	
Associates degree or some college (no degree)	6 (25.0)
Bachelor’s degree	10 (41.6)
Master’s degree	4 (16.7)
Doctoral/Professional school degree	4 (16.7)
*Employment Status*	
Employed full-time	11 (45.8)
Employed part-time	5 (20.8)
Homemaker	8 (33.4)
*Residence*	
Rural	12 (50)
Urban	12 (50)
*Race*	
Caucasian	24 (100)
*Age of child being given breastmilk*	
1–6 months	8 (33.3)
7–12 months	12 (50.0)
13–18 months	2 (8.3)
19–24 months	2 (8.3)
*Household Income*	
$25,000–$49,999	5 (20.8)
$50,000–$74,999	4 (16.7)
$75,000–$99,999	2 (8.3)
$100,000–$124,999	4 (16.7)
$125,000–$149,999	6 (25.0)
$150,000 or greater	3 (12.5)

### Definitions of healthy behaviors

The majority of mothers defined physical activity as general movement that could include structured exercise as well as walks outside or housework. Almost all mothers defined healthy eating as a balance of food groups with a concentration on fruits and vegetables while limiting sugar intake.

### Motivation for health

All of the mothers reported motivation to engage in healthy behaviors like healthy eating and physical activity. Their motivations for health centered around two main sub-themes including a desire for mental and physical self-care and to be healthy for their children.

### Mental and physical self-care

Mothers reported wanting to engage in healthy behaviors for their own personal well-being. A desire for increased energy was especially apparent among respondents. For example, “I think it’s just general well-being, usually it comes down to energy”. Another mother stated, “Honestly for me, it’s just something I can do for self-care. It’s something I can do to take time for myself, and I want to be able to chase after my kids and not feel like crap” (Mother of 5 month old). Some mothers were more interested in their physical self-care as it related to their appearance. Many reported a desire for weight loss which they felt could be supported by diet and exercise or just a general desire to have their clothes fit. One noted, “I don’t want to gain weight as I stop breastfeeding” (Mother of 4 month old).

### Children

A desire to be healthy for their children was a primary motivator among interviewees. Many women reported not only wanting to be able to stay active with their children. As one mother of an 8 month old stated;

It’s actually the zoo, being able to take my kids to the zoo and see everything they want to see and I can’t. I just don’t have enough energy to be able to do it and I have to stop. So it’s just being able to do everything within a day that I wanna get done and right now I don’t have the energy.

### Misinformation

The second overarching theme that emerged was the misinformation breastfeeding mothers received regarding how to engage in healthy behaviors. The most common topic discussed in relation to this was related to concerns about milk supply production. The theme of misinformation was demonstrated by four sub-themes; their utilization of social media, provider knowledge, knowledge through the grapevine, or a personal experience with an impact on their milk supply.

### Utilization of social media

Social media was a common source for information regarding how to engage in healthy behaviors and maintain successful breastfeeding. In particular, *Facebook* groups were often referenced as a source of information. Mothers reported being influenced by this information but at the same time not fully trusting its accuracy. As one mother stated, “With my first son I had read a blog or something, I don’t know, it probably wasn’t super evidenced-based but I read that if you work out, you’re going to lose your milk” (Mother of 9 month old). Another mother of a 7 month old reported a similar concern, “I try not to lift a whole bunch of weights just because I read on a mommy *Facebook* page or something that it’s bad. I’m not totally for sure that it’s true or not though”. Other mothers reported seeing advice on social media sites but knowing it was inaccurate. For example, “You know I think a lot of the advice I got was like really crappy *Pinterest* advice, like don’t eat this because this will happen or like don’t eat peppermint candies cause it will make you lose your milk, you know just dumb stuff like that, I don’t feel like it ever had any good advice” (Mother of 14 month old).

### Provider knowledge

A lack of accurate provider knowledge was also reported. Mothers felt lactation consultants had the knowledge needed but that their family medicine, pediatrician or obstetric physicians did not. For instance, “I’ve had to do the research on my own as to you know how to exercise and how certain diets are going to affect my breastfeeding because of where I live, the doctors aren’t very natural minded and they very much push formula” (Mother of 8 month old).

### Knowledge through the GrapeVine

Mothers had preconceived notions of how to engage in health behaviors while breastfeeding but sometimes struggled to pinpoint the sources of information. While some could recall hearing information from family or friends, many were unable to recall where this information specifically came from but just reported a general concern that had not been alleviated. For example, “I never worked out intensely before my child turned a year old because I didn’t want to impact my supply and I heard it could” (Mother of 24 month old) Another mom noted, “the only thing I know I’ve heard is just to be careful because you don’t want to lose your milk supply if you start working out and so I know that was a concern for me” (Mother of 7 month old) Some mothers reported hearing this type of information but not having it influence their personal beliefs. For instance, “it’s just like a general belief or like my sister she has said it before, she says she can’t work out if she’s exclusively breastfeeding and I mean I know that’s not true but I’ve heard it from a lot of mothers” (Mother of 11 month old).

### Needed to improve healthy behaviors

All mothers reported that steps could be taken to improve their engagement in health behaviors like physical activity and healthy eating. Within this theme two subthemes emerged, a need for greater support and access to accurate information.

### A need for greater support

Mothers indicated a need for some type of support whether that support be logistical, physical or from a provider to be able to improve their eating habits or increase their physical activity. In regard to logistical support, it was the need for childcare that was most common. For instance, “I mean obviously child care is a huge thing, if you are breastfeeding with a young child having the time do a decent workout involves changing your priorities and having help” (Mother of 4 month old). Another logistic issue mentioned was price, “I think sometimes it’s a financial issue for people to be able to purchase the food that we need to eat healthy” (Mother of 12 month old). Physical support was also mentioned, as one mother requested, “make a breastfeeding sports bra. They don’t exist and it’s ridiculous and you can’t nurse in a sports bra” (Mother of 9 month old). Finally, there was a call for more provider education and support. Specifically, women wanted their physicians to be the one providing this support. For example, “I think like some education maybe through the pediatrician or even my OB [obstetrician] would have been really nice like here are some guidelines”. Another mother of a 6 week old had a specific recommendation regarding the timing of this education and support.

There are so many times that this information can be shared. All those appointments with the doctor before baby comes, and then the day of and then at discharge, and then at a week and a week after. I think that’s just optimal time, any of those times or all of them.

### Access to accurate information

Not only did women want more support but they also wanted more access to the right information. Women felt there were too many inaccurate sources out there and they didn’t know who or what to trust when trying to determine how to exercise or what to eat. Many women just wanted everything to be housed in one place that was easily accessible. However, they were split between if resources should be housed on the internet, a brochure or a video. For example, a mother that wanted to utilize the internet stated;

I think it’s just developing something that is a one-stop shop that you kind of get all your questions answered and it’s gonna be reliable and trustworthy information. I don’t know if that exists but whenever I *Google* something I’m like Oh I shouldn’t have googled that because you get a hundred different opinions on all sides (Mother of 9 month old).

Some mothers didn’t care what the resource looked like but just that it focused on dispelling the myths surrounding engaging in healthy behaviors while breastfeeding. One mother stated, “I would like a very precise way, no beating around the bush, just exactly what’s been proven as far as how much exercise or what types of exercise could statistically deplete breast milk supply” (Mother of 9 month old). Another spoke generally, “Just having resources that dispel some of the myths would be helpful” (Mother of 7 month old).

## Discussion

This study provided vital information regarding women’s perceptions of health behaviors while breastfeeding as well as determined where future efforts should be directed to increase physical activity and improve healthy eating among breastfeeding women. Importantly, all women wanted to improve their health behaviors and were motivated to do so by either their children or for personal health benefits. This is crucial as understanding what motivates someone can help develop more effective and targeted intervention strategies [25]. These findings are also similar to previous literature that found women are motivated to be healthy if it benefits their children [11].

Despite being motivated, many women reported not engaging in healthy behaviors to the extent that they would like to. This was in part due to misinformation they had previously received either from social media or through the grapevine. This information often appeared fraught with inaccuracies and did not align with scientific recommendations. It appears women were seeking their information from these sources because they did not feel they were receiving adequate information from their healthcare provider. The most commonly reported concern was related to engagement in physical activity and a fear of milk supply reduction. As concern regarding adequate milk supply is a top reason for breastfeeding cessation, it is unsurprising that this was frequently mentioned [26]. However, the fact that this was still such a substantial issue, despite several studies consistently demonstrating that regular physical activity will not have a negative effect on the volume of milk a woman produces or its nutrient composition, is alarming [27, 28]. The translation of research findings into practice is a well-established challenge [29]. In fact, there are reports that < 20% of what physicians do have sufficient evidence to support it [30]. To improve the translation of evidence into practice more work is needed to identify the most effective strategies to tailor messaging to mothers via the appropriate medium [29]. Studies such as ours can help to determine messaging types and avenues for delivery by exploring maternal perceptions.

Although some women reported hearing about certain foods which influence milk supply, few women reported making specific dietary choices because they thought it would influence their milk supply. This is good news as other studies have shown popular myths related to maternal diet appeared to be gaining traction and influencing women’s dietary choices while breastfeeding [31]. For example, Jeong and colleagues (2017) found more than a third of breastfeeding Korean mothers were restricting certain food types that have previously been deemed appropriate nutrition sources (e.g., caffeine, spicy foods, raw foods) due to recommendations they had heard from non-medical professionals [31]. Based on our findings, American women in the Midwest appear less influenced by these dietary myths and the target area for education should be physical activity; however, further explorations regarding cultural or geographic differences is warranted. In general, efforts should continue to promote a well-balanced diet and healthy caloric intake while breastfeeding.

To address the need for better provider support, providers must first have the education and information needed to be able to disseminate accurate information to their patients. Sample strategies might include webinars or offering continuing education for healthcare providers in relation to this subject matter. Another avenue of improving knowledge and support could also include social media campaigns that come directly from hospital or clinic *Facebook* or *Instagram* accounts [32].

Finally, attempting to alter peer to peer dialogue in relation to this subject is challenging. In our study, many women recalled hearing inaccurate advice from other mothers and then this advice appeared to influence their health behavior decisions. One strategy that has proven successful in relation to breastfeeding education has been the use of peer counselors. This could involve training a group of women and then sending them out into a community to share the knowledge and information they obtained with their peers or educating existing peer breastfeeding counselors [33].

### Limitations

There were several strengths and limitations to this study that warrant discussion. The qualitative nature of this study meant the achievement of data saturation was of greater concern than number of participants thus a small sample size was obtained. The small number of participants coupled with all of the women being Caucasian and having at least a bachelor’s degree could limit the generalizability of these findings. Further, as participation was voluntary some women may have elected to discuss this topic due to individual interest in the subject matter. This study is strengthened by the geographic diversity of the sample and the rigorous qualitative analysis that was conducted. Further the average age of the child being breastfed was 8.5 months. This is considered a strength as the interviewed population included a wide range of time in which women engaged in breastfeeding (0–24 months) included a wide range of breastfeeding mothers.

## Conclusion

Breastfeeding women are motivated to eat healthy and be physically active; however, there is a lack of accurate information available to help them to do so. Many women are refraining from physical activity due to concerns related to milk supply reduction having heard issues related to this from other mothers or social media. More education materials need to be developed and disseminated to help breastfeeding women understand how to engage in healthy eating and physical activity safely and effectively. These materials should be disseminated through provider counseling at postpartum visits, children’s pediatric appointments, social media campaigns and peer education trainings. The ability to support a mother in her desire to develop or maintain healthy behaviors while breastfeeding will not only provide wide-ranging health benefits for her and her child but could result in maintenance of those health behaviors for years to come.

## Data Availability

The datasets used and/or analyzed during the current study are available from the corresponding author on reasonable request.

## Abbreviations

SDT: Self-determination Theory
